# Whiplash Syndrome Reloaded: Digital Echoes of Whiplash Syndrome in the European Internet Search Engine Context

**DOI:** 10.2196/publichealth.7054

**Published:** 2017-03-27

**Authors:** Michael Noll-Hussong

**Affiliations:** ^1^ Department of Psychosomatic Medicine and Psychotherapy University of Ulm Ulm Germany

**Keywords:** search engine, whiplash injuries, legislation & jurisprudence, medicolegal aspects, compensation and redress, compensation, accidents, traffic, adult, female, humans, incidence, insurance claim reporting, male, neck pain, prognosis, search engine analytics, whiplash syndrome, Google Trends

## Abstract

**Background:**

In many Western countries, after a motor vehicle collision, those involved seek health care for the assessment of injuries and for insurance documentation purposes. In contrast, in many less wealthy countries, there may be limited access to care and no insurance or compensation system.

**Objective:**

The purpose of this infodemiology study was to investigate the global pattern of evolving Internet usage in countries with and without insurance and the corresponding compensation systems for whiplash injury.

**Methods:**

We used the Internet search engine analytics via Google Trends to study the health information-seeking behavior concerning whiplash injury at national population levels in Europe.

**Results:**

We found that the search for “whiplash” is strikingly and consistently often associated with the search for “compensation” in countries or cultures with a tort system. Frequent or traumatic painful injuries; diseases or disorders such as arthritis, headache, radius, and hip fracture; depressive disorders; and fibromyalgia were not associated similarly with searches on “compensation.”

**Conclusions:**

In this study, we present evidence from the evolving viewpoint of naturalistic Internet search engine analytics that the expectations for receiving compensation may influence Internet search behavior in relation to whiplash injury.

## Introduction

**Figure 1:**
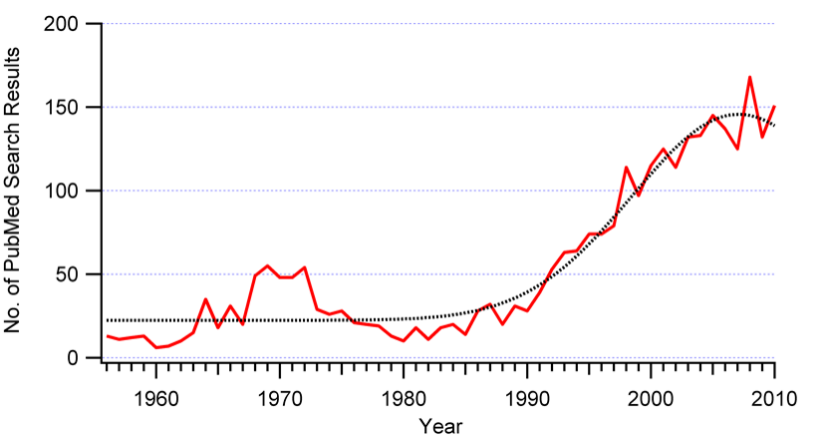
Number of publications on "whiplash" or "whiplash associated disorders" cited in Pubmed during the period from 1956 to 2010.

In many Western countries, after a motor vehicle collision, those involved seek health care for the assessment of injuries and for insurance documentation purposes. In contrast, in many less wealthy countries, there may be limited access to care and insurance may only be available to the wealthy. Against this background, the “whiplash syndrome” (ICD-10: S13.4) has been one special focus of continuous and controversial scientific research since the 1950s [1-5] (Figure 1) as the worldwide incidence of such injuries varies enormously 16-2000 per 100,000 population and the late whiplash syndrome in these cases varies between 18% to 40% [6]. Whiplash injuries are estimated to cost European society up to 10 billion euro per year [7]. Recently, and after extensive evaluation of over 1600 publications about whiplash listed in *Pubmed* [8] since 1996, the nosology of the chronic whiplash syndrome has been still classified as “doubtful” [9].

Now, 2 decades after Schrader et al’s important work in *The Lancet* showing that late whiplash syndrome after a motor vehicle collision is rare or uncommon in Lithuania [10], and Cassidy et al’s conclusion in the New England Journal of Medicine that “the elimination of compensation for pain and suffering is associated with a decreased incidence and improved prognosis of whiplash injury” [11], a new method of examining different types of “contagiosity” of diseases has evolved using the Internet search engine analytics [12-15].

According to Wikipedia, search analytics “is the use of search data to investigate particular interactions among Web searchers, the search engine, or the content during searching episodes. (...) Search analytics includes search volume trends and analysis, reverse searching (entering websites to see their keywords), keyword monitoring, search result and advertisement history, advertisement spending statistics, website comparisons, affiliate marketing statistics, and multivariate ad testing” [16]. The Internet usage especially in some European countries is presented in Table 1 [17].

**Table 1:** Internet usage on November 30, 2015.

Internet usage	Internet users	Penetration rate (% population)
Worldwide	3,366,261,156	46.37
Europe	604,147,280	73.54
Switzerland (CH)	7,180,749	87.18
Germany (DE)	71,727,551	88.36
Finland (FI)	5,117,660	93.53
France (FR)	55,429,382	83.82
Greece (GR)	6,834,560	63.21
Lithuania (LT)	2,399,678	82.15
United Kingdom (UK)	59,333,154	91.61

The number of Europeans using the Internet to obtain health information is significantly growing in all age groups, but there is especially strong growth among young women [18,19]. Individuals presented with chronic pain associated with whiplash injury are more likely to be female [20]. Internet search queries are exhaustively cataloged for marketing purposes by search engine providers [21]. Thus, as a “side-effect,” an analysis of Internet search queries can also “detect” public interests in infectious (eg, influenza) [22] and noninfectious [23,24] diseases. In addition to gathering epidemiologic data on disease incidence and prevalence through traditional, labor-intensive processes involving large surveys, chart reviews, prospective studies, or extraction from previously created databases, Internet search trend analysis tools, since they provide self-reported information by consumers, represent a complementary source of information on a population level [25,26]. The subjectively perceived “anonymity” in using the most popular organic Internet search engine may be attractive to consumers because some diseases are burdened with a social stigma [27] or are controversial and linked to monetary [28] or secondary gain [29]. Thus, Internet search data may reduce selection bias in some aspects, even though it is equally challenging to confirm the source. At the very least, using an Internet tool, culture-related attributions can be mapped on a global population level [30].

Thus, this source has the potential to reveal epidemiologic trends and patterns in near real time and with minimal expense. The current leading Internet search engine provider is owned by Alphabet Inc (marketed as Alphabet), Google Inc, which is also the brand name of the most visited website worldwide [31]. This information is freely provided to the public through Google Trends. It is of note that globally there are at present more than 3.5 billion Google searches per day and 1.2 trillion searches per year worldwide [32].

In terms of the controversial whiplash syndrome, countries in which there is an established compensation system for whiplash injury might be expected to have more Internet traffic and volume regarding whiplash injury than in countries without an established compensation system. In other words, “diverse assessments and principles for approving a claim are reflected in the fact that the prevalence of chronic spine pain after whiplash injuries (late whiplash syndrome) varies between 16% and 71% in different countries, and the proportion of whiplash injuries involved in petitions for compensation differs greatly across Europe” [33] as France and Finland [34] have the lowest and Great Britain the highest incidence of minor cervical spine trauma (eg, United Kingdom 75%, Germany 47%, Switzerland 33%, Finland 8.5%, and France 3% of all personal injuries), whereas in Greece and Lithuania whiplash injury is reported to be an almost nonexistent condition [6].

Regarding the latter, Obelieniene et al state, “Lithuania is a country in which there is no or little awareness or experience among the general population of the notion that a whiplash injury may cause chronic pain and disability. Accident victims with acute symptoms from rear end collisions generally view this as a benign injury not requiring any medical attention” [35]. Thus, it has been “hypothesized that cultural [36] and psychosocial [37-39] factors may be important in explaining why accident victims in some other societies report chronic symptoms. Such factors may include expectation of disability, symptom amplification as a result of this expectation, the effects of inappropriate therapy, insurance [40], and attribution of symptoms from nonaccident related causes (spontaneous symptoms, occupational symptoms, symptoms before an accident being amplified after an accident)”[35].

As a pilot effort, this paper deals with Google-based Internet search engine statistics on the search for “whiplash syndrome” in European countries to offer insight into both the method of Internet-based population epidemiology in whiplash-associated disorders, and the condition itself in the context of various pain cultures and national social insurance or compensation systems [41]. Specifically, the first purpose of this infodemiology or infoveillance study was to compare the Internet search patterns in Germany and the United Kingdom, countries with established compensation systems for whiplash injury, to those used in Greece and Lithuania, countries where a system for monetary compensation for motor vehicle collision injury has not yet been established. Second, in order to validate our data, we looked for the European countries for which the lowest incidence of minor cervical spine trauma has been described, that is, Finland and France [6]. Third, we compared the “googled” whiplash data with Internet search patterns concerning frequent or traumatic painful injuries; diseases and disorders like arthritis, headache, radius, and hip fracture; depressive disorders [42]; and fibromyalgia. Finally, we wanted to test if there were hints that search engine usage may reflect national changes in the medicolegal compensation rules as has been shown for Saskatchewan, Canada, where the tort-compensation system for traffic injuries, that includes payments for health and suffering, was changed to a no-fault system in 1995, which did not include such payments, resulting in a decreased incidence and improved prognosis of whiplash injury [11]. As publicly available records of search engine analytics in Google start with 2005, we chose a similar event in Switzerland; the federal court abridged the possibilities for receiving a disability pension after whiplash injury in August 2010 [43] (modified again in 2015: DFR - BGer 9C_492/2014; 03.06.2015). In this context it is interesting to know that Switzerland has the highest expenditure per claim at an average cost of €35,000 compared with the European average of €9,000, and there are large differences between German-speaking and French- or Italian-speaking parts of Switzerland [6].

## Methods

### Internet Search Engine Analytics via Google Trends and the Whiplash Syndrome

In order to compare the pattern of the Internet usage surrounding whiplash injury and compensation in Germany, Finland, France, Greece, Lithiania, and the United Kingdom, we first used Google Trends to search for uncapitalized national lay terminologies related to whiplash syndrome, such as the English word “whiplash injury,” the Finnish equivalent “piiskansiima” or “piiskaniskuvammoilta vammoja,” the French equivalent “coup de fouet (cervicale)” or “coup du lapin,” the German equivalent “schleudertrauma,” the Greek “αυχενικού τραυματισμού,” and the Lithuanian “Bicz žalos” or “kaklo nyris” or “kaklo slankstelių trauma,” and, for a more lenient “threshold,” “kaklo skausmas” (neck pain). Then we searched for the term “injury compensation” (and the respective appropriate translations and back-translations with native speakers) in Germany, Finland, France, Greece, Lithuania, and the United Kingdom.

Then, to obtain an impression of whether or not overall Internet usage in Europe for health information was different in Finland, France, Greece, and Lithuania in general, we repeated this language-adapted assessment of Google usage for arthritis (a condition known to be associated with chronic pain) in all mentioned countries, to determine how searches for this term may have differed among Germany, France, Greece, Lithuania, and the United Kingdom. In Northern Europe, the incidence of rheumatoid arthritis, the clinically most relevant subtype of arthritis, is estimated at 20-50 cases per 100,000 population and the prevalence at 0.5-1.1%, lower incidences and prevalence have been reported in Southern Europe [44]. Moreover, as more than 50% of community-dwelling adults in Europe indicate that they suffer from headache in general during the last year or less, with most headaches more prevalent in women [45], we searched for “headache” (ICD-10: R51). Important traumatic events apart from whiplash injury are hip fracture (ICD-10: S72) and radius fracture (ICD-10: S52), which are a major public health problem in the elderly and the active younger adults, respectively [46-50]. Having said this, the incidence rates of hip fracture vary from northern to southern Europe, with the highest levels in Sweden and Norway and the lowest in France and Switzerland. The reported age-standardized annual incidence rate of hip fracture, for example, in Switzerland is 346/10,000 and 137.8/100,000 in women and men, respectively [51]. The incidence of distal radius fracture is in patients >35 years of age 0.37% in females, 0.09% in males.

Finally, we searched for “depression” (ICD-10: F32 and F33) and “fibromyalgia” (ICD-10: M97) [52-54]. According to the World Health Organization (WHO), “depression is a common mental disorder. Globally, an estimated 350 million people of all ages suffer from depression. Depression is the leading cause of disability worldwide and is a major contributor to the overall global burden of disease” [55]. The 12-month prevalence of major depression is estimated as 6.9% among all Europeans [52]. Fibromyalgia is a disorder both characterized basically by chronic widespread pain and mental symptoms like fatigue, cognitive disturbances, and other symptoms, and likewise controversially or dogmatically or ideologically discussed as (late) whiplash injury. Thus, fibromyalgia appears to be a common condition in most European countries affecting up to 2% of the general population [56-60].

The Web browser we used was Mozilla Firefox 11ff [61]. The search window in Google Trends was, where not otherwise stated, restricted to the 6-year time period between January 1, 2005 and December 31, 2010: during 2011, Google updated the categorization taxonomy and modified their geographical assignment, which may lead to contorted results — therefore we skipped the year 2011 for this analysis. Moreover, we did not want to include ongoing Google searches in 2012, as this would change the data, although we cannot guarantee that Google will or has changed the Google Trends algorithms that would affect analysis of the retrospective data shown here. Finally, all of the data presented here can be most easily validated by individually using Google Trends repeating our analysis. With regard to data consistency, all data was obtained in Germany starting in March 2012 with at least annual verifications ending in August 2016, always with continuous consistent results for the time period specified previously.

### Technical Background of Internet Search Engine Analytics via Google Trends

The current world-leading search engine provider, Google Inc, provides, since August 2008, a publicly free available Internet search analytics tool based on Google search queries currently named Google Trends until September 27, 2012, known as Google Insights for Search (GIS) [62]. Google, in each case in its nationalized version, is, if uncensored, the most visited website internationally, with by far the highest market share in the search engine market in Europe [63]. For more information in the audience demographics for Google in each of mentioned countries see, for example, Alexa Internet, Inc [64].

According to information on the Google Trends website, one “can explore ‘trending stories’ in real time by category and location” using the Google Trends homepage [65]. In the appropriate frequently asked questions (FAQ) section, Google elaborates:

A trending story is a collection of Knowledge Graph topics, Search interest, trending YouTube videos, and/or Google News articles detected by our algorithms. Trending Stories rely on technology from the Knowledge Graph across Google Search, Google News, and YouTube to detect when topics are trending on these three platforms. The Knowledge Graph enables our technology to connect searches with real-world things and places. The algorithm for trending stories groups topics together that are trending at the same time on Google News, Google Search, and YouTube and ranks stories based on the relative spike in volume and the absolute volume of searches. (...) Google Trends analyzes a percentage of Google web searches to figure out how many searches were done over a certain period of time. Trends only analyzes data for popular terms, so search terms with low volume appear as 0, eliminates repeated searches from the same person over a short period of time, and filters out queries with apostrophes and other special characters. (...) Google Trends adjusts search data to make comparisons between terms easier. Otherwise, places with the most search volume would always be ranked highest. To do this, each data point is divided by the total searches of the geography and time range it represents, to compare relative popularity. The resulting numbers are then scaled to a range of 0 to 100. Data is relative across regions, i.e., just because two regions show the same number of searches for a term doesn't mean that their total search volumes are the same.

The calculation of search numbers is performed using the spelling, exactly as entered, and appropriate language [66] for Google search queries over a given period of time. The data do not contain personal information. The Internet protocol (IP) addresses of the protocols establish an educated guess on the search origins. Google elaborates that the data are normalized by dividing the datasets by a common variable to remove the effect of that variable on the data. This normalization allows a comparison of the underlying dataset characteristics. Thus, this tool does not provide absolute numbers of searches but rather a relative estimation based on search activity for the time period under study [67]. The analysis can compare 5 search terms simultaneously. If Google displayed the absolute rankings, data from regions generating the most search volume would always be ranked high (for details see [22,68]). Wikipedia states that query analysis in the context of geographical and temporal parameters produces so-called “vectors,” which may partially represent the life- and interest-space of the respective searchers [69]. Separate searches in a common context are feasible in many cases, which provide more differentiated vectors. Informative relationships and common motivators can be determined using parallel search volumes and cross-comparisons, which may be profitable as forecasts and may be retrospectively instructive for both research and marketing.

“Top searches” are search terms with the most significant levels of interest. Google states: “These terms are related to the term you’ve entered; if you didn’t enter in a search term, the top searches will be related to the category or country/territory you’ve chosen. Google determines relativity by examining searches that have been conducted by a large group of users preceding the search term you’ve entered, as well as after.”

Furthermore, as “Insights for Search examines the past values for the terms you’ve entered, it can extrapolate the future values, creating a forecast of search trends for those terms. This prediction model doesn’t take into account the context of the search term or its category, nor does it account for any business cycles that may be driving a specific market (for details, see [70]).”

Finally, Google warns on their website that the analytical data provided “aims to provide insights into broad search patterns. Several approximations are used to compute these results. The Insights for Search (or Google Trends) map is intended for general analysis of volume patterns. Borders are an approximation and may not be accurate.” Thus, Google releases its own data only in an aggregated way and often without assigning absolute values, such as the number of visitors to its graphs.

## Results

### Main Results

A comparison of the normalized data for the countrywide Google searches revealed that Google top searches for whiplash injury in Germany and the United Kingdom showed sufficient search volume and were frequently accompanied by searches for “compensation.” In other words, the concatenation of national search interest between these 2 topics such as “whiplash injury compensation” or its German counterpart “schmerzensgeld schleudertrauma,” was apparently common (see Multimedia Appendix 1). The top searches for whiplash injury in the “health” and “law and government” category for Germany and the United Kingdom can be found in Multimedia Appendix 1, respectively. Searching for “whiplash” (without “injury”) in the United Kingdom revealed the following top searches (spelling not corrected), where 18 out of 47 or ~38% of the top search results were at least semantically associated with *compensation* (Table 2).

**Table 2:** Google Trends-ranked “top searches” for “whiplash” in the United Kingdom.

Rank (#)	Top searches
1	symptoms whiplash
2	whiplash injury
3	*compensation*
4	*compensation* whiplash
5	whiplash *claim*
6	whiplash injuries
7	accident whiplash
8	miss whiplash
9	whiplash *claims*
10	neck whiplash
11	*compensation* for whiplash
12	symptoms of whiplash
13	*claim* for whiplash
14	iron man whiplash
15	car accident whiplash
16	whiplash injury *compensation*
17	whiplash treatment
18	what is whiplash
19	whiplash *payout*
20	whiplash injury *claim*
21	neck pain
22	whip lash
23	whiplash injury symptoms
24	whiplash lyrics
25	whiplash injury *claims*
26	whiplash neck injury
27	neck injury
28	*claims* for whiplash
29	whiplash trash
30	whiplash *claiming* whiplash
31	whiplash scooter
32	whiplash effects
33	average whiplash *payout*
34	whiplash marvel
35	accident *claims*
36	whiplash guidelines
37	whiplash symptoms
38	symptoms for whiplash
39	whiplash monkey
40	whiplash *payouts*
41	whiplash braid
42	Berkley whiplash
43	average whiplash *claim*
44	treatment for whiplash
45	whiplash syndrome
46	*compensation* calculator
47	whiplash *compensation* uk

The mentioned combination of Google users search interest for “whiplash” with “compensation” was not detected in the nationalized search queries in Finland (“not enough search volume to show graphs”), France (“top searches: not enough search volume to show results”), Greece (“not enough search volume to show graphs”), or Lithuania (“not enough search volume to show graphs”).

Searching for “compensation” under the Google category “health” in the United Kingdom revealed that “whiplash” and “whiplash compensation” where ranked third and fourth under top searches, after “injury compensation” (rank 1) and “compensation act” (rank 2). Searching in the United Kingdom for “compensation” in “all categories” revealed “whiplash” for the first time at rank 14, and searching for “compensation” in “law and government category” revealed “whiplash” at rank 8 (Multimedia Appendix 1). Searching for “injury compensation” in the Google category “health” in the United Kingdom revealed “whiplash injury” at rank 1 of concatenated top searches (Multimedia Appendix 1).

Looking for analog searches in “all categories” in Germany for “schmerzensgeld” (injury compensation) shows that “schleudertrauma” (whiplash injury) is at rank 3 of “top searches” (Multimedia Appendix 1). The differing distribution of “whiplash” in the different Google categories with respect to Germany and the United Kingdom may be due to categorization inconsistencies by Google.

However, no similar correlation could be detected for Lithuania searching for “atlyginimas už kūno sužalojimą,” “žalos atlyginimas,” or “žalos kompensacija,” for “kipuraha+vamman korvaukset” (injury compensation) in Finland or “αποζημίωση τραυματισμών” in Greece, as these searches revealed “not enough search volume to show graphs.” Searching for “indemnisation+dommages et intérêts” in France revealed the following results: “indemnisation chomage, chomage, indemnisation assedic, indemnisation accident, indemnisation maladie, indemnisation assurance, accident travail indemnisation, chomage partiel, indemnisation chomage partiel, assedic indemnisation chomage, indemnisation pole employ, indemnisation licenciement, accident du travail, (...).”

### Comparison With Other Diseases

Checking for top searches in all categories for “arthritis” in Switzerland (German-speaking part of Switzerland) and Germany, “niveltulehdus” in Finland, “arthrite” in France, “αρθρίτιδα” in Greece, “artritas” in Lithuania, and “arthritis” in the United Kingdom revealed the results shown in Multimedia Appendix 1. Searching for arthritis, the Internet users were transnationally most interested in terms like arthritis symptoms or arthritis treatment. As an aside, we did not find a concatenation of top searches or rising searches of arthritis with compensation in any of the 3 countries.

Checking for top searches for “headache” in the United Kingdom, “kopfschmerzen” in Switzerland and Germany, “päänsärky“ in Finland, “mal de tête ” in France, “πονοκέφαλο” in Greece (“not enough search volume to show graphs”), and “galvos skausmas” in Lithuania (“not enough search volume to show results”) revealed the results presented in Multimedia Appendix 1.

Checking for top searches in all categories for “hüftfraktur” (hip fracture) were done for Switzerland (German-speaking part of Switzerland) and Germany (both: “not enough search volume to show graphs”), “fracture de la hanche” in France (“not enough search volume to show results”), “lonkkamurtuman” in Finland (“not enough search volume to show graphs”), “κάταγμα ισχίου” in Greece (“not enough search volume to show graphs”), “šlaunikaulio lūžis” in Lithuania (“not enough search volume to show graphs”), and “hip fracture” in the United Kingdom (ranked geographic information system [GIS] top searches in the United Kingdom: “fracture of hip, hip fractures, neck of femur, hip replacement, hip fracture database, hip pain, hip fracture treatment, fractured hip, hip fracture guidelines, hip fracture classification, and hip fracture management”).

We also checked the top searches in all categories for “radius fracture” in the United Kingdom, “radiusfraktur” in Switzerland and Germany, “säde murtuma” in Finland (“not enough search volume to show results”), “fracture du radius” in France, “ακτίνα κάταγμα” in Greece (“not enough search volume to show results”), and “spindulys lūžis” in Lithuania (“not enough search volume to show results”); Multimedia Appendix 1.

We checked for top searches in all categories for “depression” in Switzerland, Germany, France, and the United Kingdom (same spelling in mentioned countries), “masennus” in Finland, “κατάθλιψη” in Greece, and “depresija” in Lithuania (we did not rule out the economic meaning of this term choosing the “health” category because this category isn’t, eg, available for Lithuania) and the results are shown in Multimedia Appendix 1. Searching for depression, the Internet users were transnationally most interested in terms like “depression symptoms” or “depression tests” (Multimedia Appendix 1). As an aside, we did not find a concatenation of top searches or rising searches with “compensation” in any of the 3 countries.

We checked for “fibromyalgia” in the United Kingdom, Finland, and France, “fibromyalgie” in Switzerland and Germany, “ινομυαλγία” in Greece (“not enough search volume to show graphs”), and “fibromialgija” in Lithuania (“not enough search volume to show graphs”). Remarkably, no concatenation of “fibromyalgia” and “compensation” (or their respective translations) could be found (Multimedia Appendix 1).

**Figure 2:**
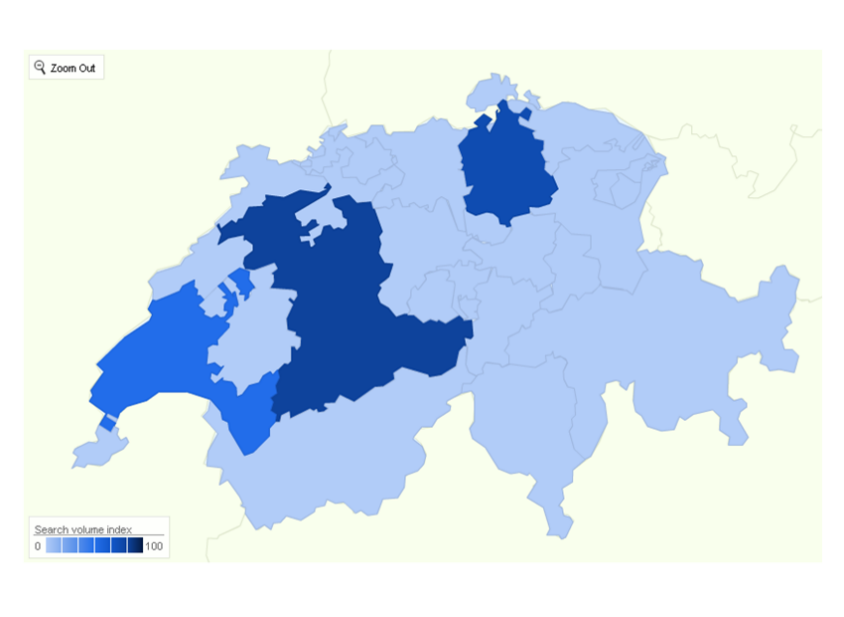
Google Insights Screenshot of cumulative regional interest for “schleudertrauma+coup de fouet cervicale+coup du lapin+colpo di frusta” in Switzerland during the period from 2005 to 2010. Regional Interest (search volume) was highest in 1. Bern, 2. Zurich and 3. Vaud. Left Upper Inset: The search interest for mentioned search terms declined ~ 40% comparing the years 2007-2009 (mean: 33) with 2010-2011 (mean: 20).

Searching for “schleudertrauma+coup de fouet cervicale+coup du lapin+colpo di frusta” (German, French, and Italian search term) in the health category for Switzerland for the years 2007-2009 and 2010-2011, respectively, revealed a decline in Google search queries for “whiplash” (Figure 2).

### Supplementary Notes

Searching for the number of advertisers for the respective national queries for “whiplash” on Alexa—the leading provider of global Web metrics—revealed that attorneys offering their assistance in law suits on personal injuries are among the top 5 in search ads for “whiplash” searches on major search engines in Germany and the United Kingdom, but not Lithuania [64].

Searching for “whiplash injury” on YouTube [71], which also belongs to Google, Inc, shows that there are about 43,700 unfiltered results (as on November 27, 2016), in which the top videos are more or less advertisements for nonevidence-based methods of “treatment” (in particular, showing the “benefits” or “secrets” of chiropractic care) and “whiplash injury compensation.”

## Discussion

### Principal Findings

This study shows that, in general, Lithuanians, Greeks, and Finns use the Internet to search for health information on conditions such as arthritis and depression in much the same way as do those from Germany, the United Kingdom, and Switzerland. However, there is a marked difference in the patterns of searches for whiplash injury or similar terms in the two former countries and Germany, the United Kingdom, and Switzerland, countries known to have high compensation rates for whiplash injury. Searches in Germany, the United Kingdom, and Switzerland for whiplash are high ranking when one examines searches combined with terms like “compensation.”

One main result is that the aforementioned combination of Google searches reflecting combined consumer interests in “whiplash injury” and “compensation” was not detected in Lithuania and other European countries (Finland, France, Greece) where cultural and psychosocial factors, including expectations, and insurance systems, have been described as significantly different from countries in which the problem of chronic whiplash is highly prevalent [72]. Actually, in Lithuania there is no formal compensation system for late whiplash injury pain and suffering, and this fact may, amongst others, influence the coping styles of the respective persons concerned [73]. Moreover, our findings reflect the low incidence of late whiplash in Finland [6,34], where total socioeconomic costs are estimated as about 1.5 million euro per annum, France [6,74], and Greece [75,76]. There has been a 70% rise in motor insurance injury claims over the 6 years leading up to 2012 in the United Kingdom, despite a 23% drop in the number of casualties actually caused by road accidents—and whiplash accounted for 70% of the total. That amounted to roughly 554,000 whiplash claims from 2010-11, that is more than 1500 claims a day. The whiplash injury costs in the United Kingdom are approximately 4.6 billion euro per annum [77]. In Germany, whiplash injury is number 1 of consequences after vehicle accidents with about 20,000 cases per year, and costs the insurance companies at least 500 million euro per annum, “official” compensation for pain and suffering due to whiplash is about 2000 euro (higher regional court (OLG) Frankfurt VRS 90, 254).

These different signs synoptically suggest that a biopsychosocial [78] understanding of chronic whiplash is important [79], especially in the “social” aspect, and the Internet is a social medium. Despite many years of research, the evidence regarding unquestionable risk factors for late whiplash is sparse but seems to include personal, societal, medicolegal, and environmental factors [80]. Against this background one should also mention an experimental study in 2001 in which participants were placed in a stationary vehicle with a curtain blocking their rear view, and exposed to a simulated rear-end collision [81]. Twenty percent of patients had symptoms at 3 days, despite the fact that no collision actually occurred [38].

Until now, the Internet search statistics [82] and social media [83] in medicine are mainly used for outbreak detection and/or the monitoring of transmissible [22,68,84,85], whereupon noninfectious diseases noticeably gain attention (eg, [86,87]). Google Insights for Search (now: Google Trends), initially developed by Google’s research and development center in Israel, is the most important freely available application on the World Wide Web. These systems are growing, and they provide multifaceted information concerning old and emerging disorders [88]. This intrinsic “predictive power” is associated with the phenomena of “swarm intelligence” [89] and typical, sometimes enigmatic properties of “social networks” [90]. However, the impact and reliability of these systems on medical and public health and individual physicians is not certain [91]. Information overload [92] in times of “Health 2.0” [93], incorrect reports (as has been shown, eg, for psychological trauma-related [94]), or Web-based information on low back pain [95]), the lack of signal specificity [96], information filtered by Internet search engine providers (ie, economic [97], political or social search engine bias) [98], media or marketing [99] interest, differing search strategies (eg, [100]), misspellings, Internet availability and local specialties [101,102], age-related differences in the accuracy of Web query-based predictions [103], seasonal effects [104], problems with incidence peaks [82,105], the unforeseeable or undisclosed evolution of search algorithms or models [106], noise [107], and (last, not least) statistical issues concerning the analysis of time series [108,109] are among the manifold confounding factors that may interfere with the development and reliability of the Internet search engine analytics, even in the medical sector [91,110]. Moreover, and from a more clinical point of view, Web-based information gathering may foster greater patient engagement in health maintenance and care [111]. Conversely, there is a relationship between searching for health information on the Web and health anxiety, a phenomenon recently named ”cyberchondria,” which may inversely influence the health of the respective Internet searchers [112,113]. Against this background, we may have to develop feasible models and tools for consumers to assess and filter health information on the Internet [114]. It is of note that other societal factors appear to also play significant roles in the rate of development of late whiplash disorder as it has been shown that, at least within Canada, regions with similar compensation systems have large differences in rates occurrence [11].

### Perspective

Future investigations will deepen our knowledge in the growing field of search engine analytics as kind of infodemiology [85,115] (or “i-epidemiology”) of the worldwide social network named Internet. Google Correlate [116], for example, enables one to find queries with a similar pattern to a target data series. The target can either be a real-world trend that one provides (eg, a dataset of event counts over time) or a query that one enters. In this context, Google Correlate uses Web search activity data to find queries with a similar pattern to a target data series. The results can be viewed on the Google Correlate website or downloaded as a comma-separated values (CSV) file [117] for further analysis. In other words, Google Correlate is like Google Trends in reverse. With Google Trends, one types in a query and receives a data series of activity. With Google Correlate, one enters a data series (the target) and receives a list of queries with a data series that follows a similar pattern.

These investigations should include the evolving impact of other social media, such as “Google+,” “Facebook” (eg, [118]), “YouTube” [119], “Wikipedia” [120], “Twitter” [121-124], and “IBM Watson” [125-127] on public health and reflect on the dark sides of the aforementioned developments, for example in terms of the possible impact of search engine analytics (on the companies behind them) on our privacy (eg, [128,129]). Second, the evolving contagiosity of ambient awareness, that is “awareness created through regular and constant reception, and/or exchange of information fragments through social media” (see [130]) has been neglected in public health thus far and could be a new form of “viral environment” for the upcoming generation. Finally, successful communication among health care providers and their patients from various sociocultural backgrounds depends on developing awareness of the normative cultural values of patients, how concepts of health and disease develop [131], and how these differ.

## Appendix

Multimedia Appendix 1 Supplementary tables S1 - S11.

## Abbreviations

CSV: comma-separated values
FAQ: freaquently asked questions
GIS: Google Insights for Search
IP: Internet protocol
