# Supplementary material for: Whiplash Syndrome Reloaded: Digital Echoes of Whiplash Syndrome in the European Internet Search Engine Context
Source: JMIR Public Health Surveill. 2017 Mar 27;3(1):e15. doi: 10.2196/publichealth.7054 (PMC5387115; doi:10.2196/publichealth.7054)
Supplement: Multimedia Appendix 1 [file publichealth_v3i1e15_app1.pdf]

Table S1. Ranked (#) ´Top searches´ in Google for “whiplash injury” or “schleudertrauma” in UK and DE. SI = Scaled Interest by GIS

#	Top searches for whiplash injury in UK	SI	Top searches for schleudertrauma in DE	SI
1	injury compensation	100	schmerzensgeld	100
2	whiplash compensation	95	schleudertrauma schmerzensgeld	90
3	whiplash injury compensation	90	hws	40
4	whiplash claim	75	symptome schleudertrauma	35
5	personal injury	65	hws schleudertrauma	35
6	whiplash injury claim	65	schmerzensgeldtabelle	20
7	whiplash symptoms	60	unfall schleudertrauma	15
8	whiplash injury symptoms	55	schmerzensgeldtabelle schleudertrauma	15
9	injury claims	50	schmerzensgeld bei schleudertrauma	15
10	whiplash claims	45	gehirnerschütterung	10
11	whiplash injury claims	45	auffahrunfall	10
12	neck injury	40	autounfall schleudertrauma	10
13	whiplash neck injury	40	schmerzensgeld hws	10
14	personal injury claim	30	behandlung schleudertrauma	10
15	personal injury claims	20	hws schleudertrauma schmerzensgeld	10
16	symptoms of whiplash	20	auffahrunfall schleudertrauma	10
17	whiplash injury payout	20	distorsion	5
18	whiplash injuries	20	schleudertrauma nach unfall	5
19	what is whiplash	20	schmerzensgeld tabelle	5
20	whiplash treatment	15	spätfolgen schleudertrauma	5
21	whiplash injury treatment	10	therapie schleudertrauma	5
22			autounfall schmerzensgeld	5
23			schleudertrauma dauer	5
24			schmerzensgeld nach schleudertrauma	5
25			schleudertrauma symptome schmerzensgeld	5

Table S2. Ranked (#) ´Top searches´ related to "whiplash injury" in different Google categories in UK

#	**Top searches for *whiplash injury* - All Categories**	**Top searches for *whiplash injury* - Health Category**	**Top searches for *whiplash injury* - Law & Government Category**
1	whiplash compensation	whiplash symptoms	whiplash injury compensation
2	whiplash injury compensation	whiplash injury symptoms	whiplash compensation
3	whiplash claim	neck injury	personal injury
4	whiplash injury claim	whiplash neck injury	whiplash claim
5	personal injury	whiplash injury compensation	whiplash injury claim
6	neck injury	whiplash compensation	whiplash claims
7	whiplash symptoms		whiplash injury claims
8	injury claims		
9	whiplash injury symptoms		
10	whiplash neck injury		
11	whiplash claims		
12	whiplash injury claims		
13	personal injury claim		
14	whiplash injuries		

Table S3. Ranked (#) ´Top searches´ related to “schleudertrauma” [whiplash injury] in different Google categories in DE

#	**Top searches for *schleudertrauma* - All Categories**	**Top searches for *schleudertrauma* - Health Category**	**Top searches for *schleudertrauma* - Law & Government Category**
1	schmerzensgeld	hws schleudertrauma	schmerzensgeld
2	schmerzensgeld schleudertrauma	symptome schleudertrauma	schmerzensgeld schleudertrauma
3	Hws		schmerzensgeldtabelle
4	hws schleudertrauma		schmerzensgeldtabelle schleudertrauma
5	schleudertrauma symptome		schmerzensgeld bei schleudertrauma
6	schmerzensgeldtabelle		hws schleudertrauma
7	unfall schleudertrauma		schmerzensgeld hws schleudertrauma
8	schmerzensgeld bei schleudertrauma		
9	schmerzensgeldtabelle schleudertrauma		
10	schmerzensgeld hws		
11	autounfall schleudertrauma		
12	hws schleudertrauma schmerzensgeld		
13	gehirnerschütterung		
14	auffahrunfall		
15	auffahrunfall schleudertrauma		
16	schleudertrauma behandlung		

Table S4. Ranked (#) ´Top searches´ in Google for "compensation" in UK

#	**Top searches for *compensation* - <u>All Categories</u>**	**Top searches for *compensation* - <u>Health Category</u>**	**Top searches for *compensation* - <u>Law & Government</u>**
1	compensation scheme	compensation act	injury compensation
2	criminal compensation	injury compensation	compensation claims
3	compensation claim	whiplash	claim compensation
4	injury compensation	whiplash compensation	criminal compensation
5	compensation claims	compensation claims	accident compensation
6	criminal injuries compensation	accident compensation	criminal injuries compensation
7	financial compensation scheme	asbestos compensation	compensation act
8	financial services compensation	nhs compensation	whiplash
9	compensation act	mesothelioma	personal injury
10	accident compensation	whiplash symptoms	whiplash compensation
11	flight compensation	mesothelioma compensation	personal injury compensation
12	Whiplash	whiplash injury	unfair dismissal
13	whiplash compensation	compensation calculator	compensation unfair dismissal
14	bank compensation	compensation recovery	injury compensation claims
15	endowment compensation	car accident compensation	compensation calculator
16	personal injury compensation		accident claim
17	compensation calculator		accident claims
18	compensation solicitors		accident compensation claims
19	unfair dismissal		redundancy compensation
20	unfair dismissal compensation		employment tribunal
21	fsa compensation		compensation lawyers
22	Fscs		compensation for whiplash
23	fscs compensation		employment tribunal compensation
24	workers compensation		crime compensation
25	criminal compensation board		compensation amounts
26	compensation recovery unit		claiming compensation
27	redundancy compensation		compensation culture
28	compensation for whiplash		compensation awards
29	claiming compensation		personal injury claims
30	airline compensation		car accident compensation
31	executive compensation		compensation act 2006
32	medical compensation		constructive dismissal
33	royal mail compensation		personal injury claim
34	bank compensation scheme		police compensation
35	flight delay compensation		victim compensation
36	fsa compensation scheme		miners compensation
37	compensation agency		land compensation act
38	criminal injury compensation		compensation payouts
39	land compensation act		constructive dismissal compensation
40	car accident compensation		whiplash injury compensation
41	compensation letter		work accident compensation
42	accident claims		whiplash injury

43	northern rock compensation		medical negligence
44	exposure compensation		medical negligence compensation
45	equitable life compensation		compensation claims uk
46	compensation and benefits		injury compensation calculator
47	Cica		injury lawyers
48	easyjet compensation		compensation order
49	bt compensation		whiplash compensation claims
50	compensation lawyers		accident at work

Table S5. Ranked (#) ´Top searches´ in Google for “injury compensation” in UK

#	**Top searches *for injury compensation* in <u>All Categories</u> – UK**	**Top searches for *injury compensation* in <u>Health Category</u> - UK**	**Top searches *for injury compensation* in <u>Law & Government Category</u> - UK**
1	personal injury	whiplash injury	personal injury
2	compensation personal injury		personal injury compensation
3	compensation for injury		compensation for injury
4	injury claim		compensation claim
5	compensation claim		injury claim
6	compensation claims		injury claims
7	injury claims		compensation claims
8	injury compensation claim		injury compensation claim
9	accident compensation		injury compensation claims
10	injury compensation claims		personal injury claim
11	criminal injury		personal injury claims
12	accident injury compensation		whiplash
13	criminal injury compensation		whiplash compensation
14	Whiplash		whiplash injury
15	whiplash injury		whiplash injury compensation
16	whiplash compensation		accident claim
17	personal injury claim		accident claims
18	whiplash injury compensation		compensation calculator
19	personal injury claims		injury compensation calculator
20	work injury compensation		injury lawyers
21	accident claim		compensation lawyers
22	compensation calculator		accident compensation claims
23	car injury compensation		personal injury solicitors
24	injury compensation calculator		back injury compensation
25	back injury compensation		injury compensation amounts
26	injury at work		personal injury calculator
27	accident claims		
28	injury lawyers		
29	injury compensation lawyers		
30	personal injury solicitors		
31	criminal injuries		
32	criminal injuries compensation		
33	injury compensation amounts		
34	personal injury calculator		
35	industrial injury compensation		
36	whiplash claim		
37	injury compensation guidelines		
38	accident at work		

Table S6. Ranked (#) ´Top searches´ in Google for “schmerzensgeld” [injury compensation] in DE

#	**Top searches for *schmerzensgeld* - <u>All Categories</u>**	**Top searches for *schmerzensgeld* - <u>Health Category</u>**	**Top searches for *schmerzensgeld* - <u>Law & Government Category</u>**
1	schmerzensgeldtabelle		schmerzensgeldtabelle
2	schmerzensgeld unfall		schleudertrauma
3	schleudertrauma		schmerzensgeld schleudertrauma
4	schleudertrauma schmerzensgeld		tabelle schmerzensgeld
5	schmerzensgeld tabelle		hws
6	Hws		adac schmerzensgeld
7	schmerzensgeld hws		hws schmerzensgeld
8	adac schmerzensgeld		unfall schmerzensgeld
9	körperverletzung		körperverletzung
10	schadensersatz		schadensersatz
11	schadensersatz schmerzensgeld		schmerzensgeld körperverletzung
12	körperverletzung schmerzensgeld		schmerzensgeld höhe
13	schmerzensgeld höhe		schadensersatz schmerzensgeld
14	verkehrsunfall schmerzensgeld		hundebiss
15	autounfall schmerzensgeld		schmerzensgeldtabelle adac
16	hundebiss		schmerzensgeld verkehrsunfall
17	hundebiss schmerzensgeld		autounfall schmerzensgeld
18	schmerzensgeld nach unfall		schmerzensgeld hundebiss
19	adac schmerzensgeldtabelle		schmerzensgeld bgb
20	bgb schmerzensgeld		urteil schmerzensgeld
21	schadenersatz		schadenersatz
22	arbeitsunfall schmerzensgeld		schmerzengeld
23	arbeitsunfall		schmerzensgeld wieviel
24	wieviel schmerzensgeld		schmerzensgeld beleidigung
25	beleidigung schmerzensgeld		schmerzensgeldkatalog
26	schmerzengeld		rechtsanwalt schmerzensgeld
27	schmerzensgeld bei unfall		schmerzensgeld verjährung
28	schmerzensgeld auffahrunfall		schmerzensgeld bei schleudertrauma
29	distorsion		auffahrunfall schmerzensgeld
30	schmerzensgeld behandlungsfehler		urteile schmerzensgeld
31	schmerzensgeld katalog		adac schmerzensgeld tabelle
32	schmerzensgeld prellung		schmerzensgeld bei körperverletzung
33	verjährung schmerzensgeld		schmerzensgeld bei unfall
34	schmerzensgeldkatalog		schmerzensgeld katalog
35	klage schmerzensgeld		hacks schmerzensgeld
36	schmerzensgeld mobbing		schmerzensgeld nach unfall
37	schmerzensgeld bei schleudertrauma		schmerzensgeldtabelle körperverletzung
38	unfallversicherung schmerzensgeld		nasenbeinbruch schmerzensgeld
39	schmerzensgeld bei körperverletzung		schmerzensgeld hws distorsion
40	schmerzensgeld nach		schmerzensgeld prellungen

	verkehrsunfall		
41	urteile schmerzensgeld		behandlungsfehler schmerzensgeld
42	schmerzensgeld nach autounfall		schmerzensgeld prellung
43	schmerzensgeld antrag		schmerzensgeldtabellen
44	hws distorsion schmerzensgeld		schmerzensgeldhöhe
45	schmerzensgeld bei hws		hws distorsion
46	nasenbeinbruch schmerzensgeld		hws schleudertrauma
47	adac schmerzensgeld tabelle		

Table S7. Ranked (#) ´Top searches´ in ´All Categories´ in Google for arthritis in CH, DE, FI, FR, GR, LT, and UK

#	**Top searches for *arthritis* - CH**	**Top searches for *arthritis* - DE**	**Top searches for *arthrite* - F**	**Top searches for *niveltulehdus* - FI Top searches for *αρθρίτιδα* - GR**	**Top searches for *artritas* - LT**	**Top searches for *arthritis*- UK**	
1	rheumatoide arthritis	rheumatoide arthritis	arthrose	reaktiivinen niveltulehdus	ρευματοειδής αρθρίτιδα	reumatoidinis artritas	rheumatoid arthritis
2	rheumatoid arthritis psoriasis	arthrose arthrite	niveltulehdus oireet			arthritis symptoms	
3	arthrose	psoriasis arthritis	arthrite rhumatoide	nivelreuma			arthritis pain
4	psoriasis arthritis	arthrose	polyarthrite				arthritis treatment
5	psoriasis	arthrose arthritis	arthrite dentaire				arthritis uk
6	arthrose arthritis	rheuma	arthrite septique				psoriatic arthritis
7	rheuma	rheumatoid arthritis	arthrite inflammatoire				arthritis care
8	arthritis pain	rheuma arthritis	arthrite genou				knee arthritis
9	juvenile arthritis	arthritis symptome	artrite				arthritis research
10	arthritis symptome	reaktive arthritis	traitement arthrite				osteoarthritis
11		rheumatische arthritis	rhumatisme				what is arthritis
12		arthritis pain	arthrite symptomes				symptoms of arthritis
13		juvenile arthritis	arthrite juvénile				arthritis diet
14		behandlung arthritis	poly arthrite				osteo arthritis
15		gicht	arthrite articulaire				arthritis hip
16		artritis	peri arthrite				rheumatoid arthritis symptoms
17		arthritis finger	arthrite chronique				septic arthritis
18		arthritis ernährung	arthrite du genou				arthritis dogs
19		arthritis treatment	arthrose et arthrite				rheumatism
20		arthritis rheumatism	arthrite juvenile				reactive arthritis

21		athritis	polyarthrite rhumatoide				treatment for arthritis
22		chronische arthritis	arthrite ankylosante				neck arthritis
23		arthritis relief	arthrite infectieuse				arthritus
24		arthritis knie	spondylarthrite				juvenile arthritis
25		polyarthritis	arthrite hanche				rheumatoid arthritis treatment
26		rheumatoider arthritis	arthrite psoriasique				arthritis relief
27		rheumatoide arthritis symptome	arthrite doigts				arthritis research campaign
28		arthritis pain relief	arthrite main				methotrexate
29		Gelenkschmerzen	arthrite enfant				inflammatory arthritis
30		lyme arthritis	arthrite rhumatoïde				psoriasis
31		yersinien arthritis	arthrite symptome				arthritis feet
32		arthritis symptoms	arthrite réactionnelle				psoriasis arthritis
33		yersinien	arthrite de hanche				arthritis drugs
34		arthritis psoriatica	arthrite poignet				rhumatoid arthritis
35		arthritis urica	arthrite chronique juvénile				arthritis cure
36		rheuma symptome	artérite				arthritis in dogs
37		was ist arthritis	arthrite cervicale				gout
38		arthritis wikipedia	arthrite des mains				dog arthritis
39		schuppenflechte arthritis	arthrite doigt				arthritis in hands
40		arthritis bh	arthrite de lyme				arthritis society
41		arthritis and rheumatism					causes of arthritis
42		psoriatic arthritis					ra
43		arthritis medicine					arthritis and rheumatism

44		atritis					arthritis in fingers
45		knee arthritis					lupus
46		athrose					glucosamine
47		therapie rheumatoide arthritis					arthritis pain relief
48		mtx					rheumatology
49		ernährung bei arthritis					arthritis remedies
50		rheumatoiden arthritis					osteoporosis

Table S8. Ranked (#) ´Top searches´ in ´All Categories´ in Google for headache in CH, DE, FI, FR, and UK

#	**Top searches for *kopfschmerzen* – CH**	**Top searches for *kopfschmerzen* - DE**	**Top searches for *päänsärky* - FI**	**Top searches for *mal de tête* - FR**	**Top searches for *headache* - UK**
1	gegen kopfschmerzen	gegen kopfschmerzen	päänsärky raskaus	maux de tête	headache symptoms
2	kopfschmerzen symptome	kopfschmerzen schwindel	migreeni	mal de tete	headaches
3	schwindel	übelkeit kopfschmerzen	huimaus	maux de tete	migraine
4	schwindel kopfschmerzen	kopfschmerzen schwangerschaft	huimaus päänsärky		migraine headache
5	Migräne	starke kopfschmerzen	kuume		tension headache
6	kopfschmerzen schwangerschaft	migräne	jatkuva päänsärky		sinus headache
7	starke kopfschmerzen	fieber	päänsärky kuume		pregnancy headache
8	Kopfweh	kopfschmerz	päänsärky pahoinvointi		pressure headache
9	ursachen kopfschmerzen	kopfschmerzen hausmittel	aivokasvain		headache nausea
10	kopfschmerzen übelkeit	ursachen kopfschmerzen	kova päänsärky		headache causes
11	Cluster	kopfschmerzen was tun	toispuoleinen päänsärky		cluster headache
12	hirntumor	kopfschmerzen augen	aivokasvain päänsärky		headache dizziness
13	kopfschmerzen augen	kopfschmerzen nacken	päänsärky ja raskaus		severe headache
14	kopfschmerzen hausmittel	müdigkeit kopfschmerzen	verenpaine päänsärky		head pain
15	cluster kopfschmerzen	nackenschmerzen kopfschmerzen	päänsärky väsymys		pregnant headache
16	müdigkeit kopfschmerzen	nackenschmerzen	lapsen päänsärky		constant headache
17	kopfschmerzen hirntumor	kopfschmerzen bei kindern	aivokalvontulehdus		sore throat
18		kopfschmerzen hinterkopf	hortonin päänsärky		sore throat headache
19		kopfschmerzen kinder	pääkipu		headache cure
20		kopfschmerzen in schwangerschaft	päänsärky takaraivossa		bad headache
21		schwanger kopfschmerzen	sarjoittainen päänsärky		blood pressure headache
22		kopfschmerzen und übelkeit	äkillinen päänsärky		chronic headache
23		kopfschmerzen stirn	päänsärky ohimolla		causes of headache
24		durchfall kopfschmerzen	päänsärky ja huimaus		headache in pregnancy
25		schwindel und kopfschmerzen	kuume ja päänsärky		stress headache
26		cluster kopfschmerzen	poskiontelotulehdus		nausea and headache
27		kopfschmerzen wetter	flunssa päänsärky		headache behind eyes
28		stechende	päänsärky hoito		headache relief

		kopfschmerzen			
29		hausmittel gegen kopfschmerzen	päänsärky ja pahoinvointi		cough headache
30		hirntumor	flunssa		brain tumour
31		kopfschmerzen ursache	niskakipu		headache tablets
32		paracetamol	lasten päänsärky		head ache
33		einseitige kopfschmerzen	jännityspäänsärky		headache and dizziness
34		paracetamol kopfschmerzen			morning headache
35		gehirntumor			stiff neck headache
36		schwindel übelkeit kopfschmerzen			migraine symptoms
37		grippe kopfschmerzen			sinusitis
38		kopfschmerzen homöopathie			headache cures
39		kopfschmerzen kind			tension headaches
40		ständig kopfschmerzen			persistent headache
41		alkohol kopfschmerzen			tension headache symptoms
42		mittel gegen kopfschmerzen			sinusitis headache
43		kopfschmerzen schläfe			exercise headache
44		schnupfen kopfschmerzen			headache virus
45		hws kopfschmerzen			temple headache
46		fieber und kopfschmerzen			meningitis
47		ständige kopfschmerzen			headache behind eye
48		hirnhautentzündung			headache remedies
49		chronische kopfschmerzen			headache in children
50		augenschmerzen kopfschmerzen			headache types

Table S9. Ranked (#) ´Top searches´ in ´All Categories´ in Google for radius fracture in CH, DE, FR, and UK

#	Top searches for *radiusfraktur* - CH	Top searches for *radiusfraktur* - DE	Top searches for *fracture du radius* - FR	Top searches for *radius fracture* - UK
1	distale radiusfraktur	distale radiusfraktur	fracture du poignet	distal radius
2		radiusfraktur handgelenk		distal radius fracture
3		radiusfraktur therapie		distal
4		distal		fracture of radius
5		ao radiusfraktur		wrist fracture
6		radiusfraktur physiotherapie		radius bone
7		distaler radiusfraktur		radius bone fracture
8		radiusfraktur klassifikation		radius and ulna
9		radiusfraktur distal		radius head fracture
10				colles fracture

Table S10. Ranked ´Top searches´ in ´All Categories´ in Google for depression in CH, DE, FI, FR, GR, LT, and UK

#	**Top searches for *depression* – CH**	**Top searches for *depression* - D**	**Top searches for *masennus* - FI**	**Top searches for *dépression* - FR**	**Top searches for *κατάθλιψη* - GR**	**Top searches for *depresija* - LT**	**Top searches for *depression* - UK**
1 symptome depression	depressionen	masennus oireet	la dépression	κατάθλιψη συμπτώματα	depresija simptomai	the depression	
2	depression test	depression test	synnytyksen jälkeinen masennus	depression	καταθλιψη	depresija testas	depression symptoms
3 Depressionen	symptome depression	masennus testi	dépression nerveuse		depresija gydymas	great depression	
4	great depression	forum depression	masennustesti	dépression symptomes			anxiety depression
5	la depression	great depression	masennus hoito	france dépression			anxiety
6	postnatale depression	manische depression	nuorten masennus	dépression test			depression help
7	manische depression	therapie depression	ahdistus	dépression définition			manic depression
8	depression wikipedia	hilfe depression	ahdistus masennus	dépression travail			depression uk
9	anxiety depression	major depression	mielenterveys	grande dépression			depression test
10	depression definition	endogene depression	depressio	forum dépression			symptoms of depression
11	Burnout	behandlung depression	lapsen masennus	soigner dépression			the great depression
12	therapie depression	reaktive depression	keskivaikea masennus	traitement dépression			post natal depression
13	burnout depression	kompetenznetz depression	keskustelu masennus	déprime			what is depression
14	forum depression	depressiv	depression	déprime dépression			anxiety and depression
15	depression symptomes	wikipedia depression	vakava masennus	dépression symptome			postnatal depression
16	Depressiv	selbsthilfegruppe depression	väsymys	signes dépression			depression treatment
17	depression hilfe	depression medikamente	stressi	maniaco dépression			clinical depression
18	depression	the great	masennus	grossesse			stress

	symptoms	depression	väsymys	dépression			
19	depression behandlung	antidepressiva	masennus raskaus	la grande dépression			signs of depression
20	the great depression	depression angehörige	masennus suomi24	dépression symptômes			nhs depression
21	antidepressiva	depression selbsthilfe	psykoottinen masennus	soigner la dépression			bipolar depression
22	endogene depression	definition depression	vaikea masennus	fatigue dépression			bipolar
23	depression was tun	bündnis depression	itsemurha	dépression post partum			mental health
24	johanniskraut	johanniskraut depression	nuoren masennus	angoisse			depressed
25	depression angehörige	depression symptoms	alkoholi masennus	dépression saisonnière			suicide
26	major depression	johanniskraut	masennuksen hoito	dépression enfant			depression medication
27		postnatale depression	masennuslääkkeet	bipolaire			nice depression
28		ursachen depression	psykoosi	dépression bipolaire			help with depression
29		schwangerschaft depression	skitsofrenia	la dépression nerveuse			pregnancy depression
30		schwere depression	lasten masennus	dépression réactionnelle			american depression
31		depression kinder	masentunut	maison de repos			st depression
32		anzeichen depression	paniikkihäiriö	signes de dépression			treatment for depression
33		große depression	masennus sairasloma	sos dépression			alcohol depression
34		bipolare depression	masennus apua	dépression chronique			causes of depression
35		alkohol depression	masennus lääkkeet	doctissimo dépression			teenage depression
36		anxiety depression	masennuksen oireet	test de dépression			dealing with depression
37		suizid	kaksisuuntainen mielialahäiriö	depression nerveuse			depression drugs
38		bündnis gegen depression	masennus wikipedia	dépression au travail			depression alliance
39		psychose	yksinäisyys	dépression masquée			depression forum
40		schizophrenie	raskauden jälkeinen masennus	depression symptomes			severe depression
41		serotonin depression	lievä masennus	info dépression			stress and depression
42		depressionen test	unettomuus masennus	grossesse et dépression			depression self help
43		hilfe bei depression	vanhusten masennus	anticyclone			depression quotes
44		depression homöopathie	masennus itsehoito	dépression majeure			coping with depression
45		serotonin	unettomuus	guérir la dépression			antidepressants
4		neurotische	psykoterapia	causes			depression

6		depression		dépression			wiki
47		burnout	epätyypillinen masennus	signe dépression			nice guidelines
48		depresion	krooninen masennus	dépression chat			nice guidelines depression
49		depression icd 10	työuupumus	vaincre la dépression			beat depression
50		icd 10	uupumus				depression statistics

Table S11. Ranked ´Top searches´ in ´All Categories´ in Google for fibromyalgia in CH, DE, FI, FR, and UK

#	Top searches for *fibromyalgie* - CH	Top searches for *fibromyalgie* - DE	Top searches for *fibromyalgia* - FI	Top searches for *fibromyalgie* - FR	Top searches for *fibromyalgia* - UK
1	symptome fibromyalgie	fibromyalgie symptome	fibromyalgia oireet	la fibromyalgie	symptoms fibromyalgia
2	la fibromyalgie	rheuma	fibromyalgia kipupisteet	fibromyalgie symptome	fibromyalgia pain
3		fibromyalgie klinik	reuma	maladie fibromyalgie	fibromyalgia uk
4		fibromyalgie forum		symptomes fibromyalgie	fibromyalgia syndrome
5		fibromyalgie therapie		traitement fibromyalgie	fibromyalgia treatment
6		fibromyalgie ärzte		fibromyalgie association	what is fibromyalgia
7		fibromyalgie tender points		fibromyalgie forum	symptoms of fibromyalgia
8		fibromyalgie rente		fibromyalgie france	fibromyalgia points
9		fibromyalgie syndrom		cure fibromyalgie	fibromyalgia support
10		selbsthilfegruppe fibromyalgie		fibromialgie	nhs fibromyalgia
11		muskelschmerzen		fibromyalgie definition	fibromyalgia causes
12		fibromyalgie ernährung		fibromyalgie diagnostic	polymyalgia
13		fibro		spasmophilie	fibromyalgia association
14		weichteilrheuma		douleurs musculaires	fibromyalgia diet
15		fibromyalgie behandlung		lyrica	fybromyalgia
16		fibromyalgia		fibromyalgie lyrica	fibromyalgia diagnosis
17		reha fibromyalgie		fatigue chronique	muscle pain
18		fibromyalgie münchen		fibromyalgie cure thermale	chronic fatigue syndrome
19		fibromyalgie wikipedia		cure thermale	fibro
20		fibromyalgie hamburg		points fibromyalgie	fibromyalgia help
21		homöopathie fibromyalgie		fibro	fibromyalgia tender points
22		fibromyalgie diagnose		fibromalgie	cfs
23		was ist fibromyalgie		association de fibromyalgie	treatment for fibromyalgia
24		fibromyalgie kliniken		fibromyalgia	fibromyalgia rheumatica
25		fibromyalgie medikamente		fibromyalgie symptômes	arthritis symptoms
26		gelenkschmerzen		fibromyalgie 2010	fibromyalgia support group
27		treffpunkt fibromyalgie		définition fibromyalgie	fibromyalgia more condition_symptoms
28		selbsthilfegruppen		polyarthrite	amitriptyline

		fibromyalgie			
29		fibromyalgie ursachen		fibromyalgie sos	myalgia
30		fibromyalgiesyndrom		fybromyalgie	polymyalgia rheumatica
31		fibromyalgie tenderpoints		cymbalta	causes of fibromyalgia
32		fibromyalgie selbsthilfe		fibromyalgie 2009	fibromyalgia forum
33		rheumaliga		points de fibromyalgie	fibromyalgia support groups
34		gdb fibromyalgie		fibromyalgie homeopathie	fibromyalgia cure
35		klinik für fibromyalgie		douleur musculaire	fibromyalgia medication
36				soigner la fibromyalgie	lupus symptoms
37				fybromialgie	lyrica
38				douleurs jambes fibromyalgie	fibromyalgia research
39				fibromyalgie 2008	fibromyalgia trigger points
40				myalgie	fibromyalgia association uk
41					fms
42					treatment of fibromyalgia
43					costochondritis
